# Association between Age of Menopause and Thickness of Crestal Cortical Bone at Dental Implant Site: A Cross-Sectional Observational Study

**DOI:** 10.3390/ijerph17165868

**Published:** 2020-08-13

**Authors:** Yi-Chun Ko, Ming-Tzu Tsai, Lih-Jyh Fuh, Min-Jia Tsai, Xuan-Hui Wang, Heng-Li Huang, Jui-Ting Hsu

**Affiliations:** 1 School of Dentistry, China Medical University, Taichung 404, Taiwan; Chip38916@hotmail.com (Y.-C.K.); ljfuh@mail.cmu.edu.tw (L.-J.F.); 2 Department of Biomedical Engineering, Hungkuang University, Taichung 433, Taiwan; anniemtt@sunrise.hk.edu.tw; 3 Department of Dentistry, China Medical University and Hospital, Taichung 404, Taiwan; b50703@gmail.com; 4 Master Program for Biomedical Engineering, China Medical University, Taichung 404, Taiwan; u108209701@cmu.edu.tw; 5 Department of Bioinformatics and Medical Engineering, Asia University, Taichung 413, Taiwan

**Keywords:** dental implants, dental cone-beam computed tomography, cortical bone thickness, menopause, fertility

## Abstract

Satisfactory host bone quality and quantity promote greater primary stability and better osseointegration, leading to a high success rate in the use of dental implants. However, the increase in life expectancy as a result of medical advancements has led to an aging population, suggesting that osteoporosis may become a problem in clinical dental implant surgery. Notably, relative to the general population, bone insufficiency is more common in women with post-menopausal osteoporosis. The objective of this study was to compare the thickness of the crestal cortical bone at prospective dental implant sites between menopausal and non-menopausal women. Prospective dental implant sites in the jawbone were evaluated in two groups of women: a younger group (<50 years old), with 149 sites in 48 women, and an older group (>50 years old) with 191 sites, in 37 women. The thickness of the crestal cortical bone at the dental implant site was measured based on each patient’s dental cone-beam computed tomography images. For both groups, one-way analysis of variance and Tukey’s post-test were used to assess the correlation between cortical bone thickness and the presence of implants in the four jawbone regions. Student’s *t*-test was further used to compare differences between the older and younger groups. From the retrospective study results, for both groups, thickness of the crestal cortical bone was the highest in the posterior mandible, followed by anterior mandible, anterior maxilla, and posterior maxilla. Compared with the younger group, the older group had a lower mean thickness of the crestal cortical bone. Among the four regions, however, only in the posterior maxilla was the crestal cortical bone significantly thinner in the older group than in the younger group.

## 1. Introduction

Osteoporosis is a severe health problem. Based on the World Health Organization’s (WHO) diagnostic criteria, osteoporosis occurs when patients have a bone mineral density (BMD) that is >2.5 standard deviations below the BMD mean value of young healthy women [1]. Such BMD is measured through dual-energy X-ray absorptiometry. Post-menopausal women have decreased estrogen levels, which severely reduces bone mass; this phenomenon is known as post-menopausal osteoporosis. According to figures reported in previous studies, approximately 30% of post-menopausal women in the United States and Europe have osteoporosis, 40% of whom will experience at least one episode of fragility fracture in their remaining lifetime [2]. In addition to higher incidence rates of hip bone, spine, and wrist fractures, studies have indicated that osteoporosis may increase the rate of tooth loss and loss of the tooth-supporting alveolar bone [3].

Dental implantation is a common treatment for loss of teeth; it involves inserting titanium alloy dental fixtures in place of the patient’s missing teeth. Because titanium has excellent biocompatibility with the human tissue, titanium alloy dental fixtures can be osseointegrated and assimilated into the alveolar bone [4]. The osseointegration rate is a critical factor determining the success of dental implantation surgery, and osseointegration capacity is influenced by the dental implant site as well as bone quality and quantity. Higher host bone quality and quantity provide greater initial stability for dental implants, resulting in enhanced osseointegration and higher long-term survival rates among patients receiving dental implants [5,6,7]. Therefore, dentists must determine the bone quality and quantity of the patient’s jawbone prior to performing dental implant surgery, and dental cone-beam computed tomography (CBCT) has become an increasingly popular method for such examinations [8]. In general, jawbone quality and quantity are indicated by cortical bone thickness and cancellous bone density, respectively. However, most studies have investigated BMD [9,10,11,12], neglecting the thickness of the crestal cortical bone of the dental implant site [13,14,15].

The objective of this study was to evaluate the association between being of menopausal age and the thickness of the crestal cortical bone at the prospective dental implant site for the female population by using dental CBCT images. The study findings can serve as a reference for dentists prior to their performance of implantation surgery.

## 2. Materials and Methods 

### 2.1. CBCT Examinations of Patients and Implant Sites

CBCT images were collected from 85 female patients (mean age: 46.0 ± 14.3 years) who had undergone a dental implant placement at any time between 2013 and 2016. The patients were assessed by dentists, and all dental implants were placed at healed sites on the jawbone. The exclusion criteria were as follows: (1) having artifacts due to metal content (amalgam, orthodontic bracket, or miniscrew), (2) having motion artifacts due to head movement, and (3) having undergone bone graft surgery at the prospective dental implant site. CBCT scans were preformed 2 weeks prior to dental implant surgery. The CBCT machine (AZ 3000; Asahi Roentgen, Kyoto, Japan) was used with the following technical parameters: 85 kV, 3 mA, and a voxel resolution of 150 µm. Prior to undergoing CBCT, each patient was asked to insert radiopaque gutta-percha indicators, which enabled researchers to precisely determine the position of the dental implant site on the CBCT images. The menopause status of the 85 patients could not be determined because of the retrospective nature of this study. Therefore, the patients were divided into two groups: patients younger than the average menopause age (<50 years old; 48 patients with a mean age of 35.6 ± 9.6 years; ranging from 19 to 49 years) and patients older than the average menopause age (≥50 years old; 37 patients with a mean age of 58.5 ± 7.3 years; ranging from 50 to 79 years). The patients’ CBCT images were used to identify implant sites. For the younger group, 149 prospective dental implant sites were identified—16 in the anterior mandible, 26 in the anterior maxilla, 59 in the posterior mandible, and 48 in the posterior maxilla. For the older group, 191 prospective dental implant sites were identified—24 in the anterior mandible, 19 in the anterior maxilla, 64 in the posterior mandible, and 84 in the posterior maxilla. This retrospective study was approved by the Institutional Review Board of China Medical University Hospital (CMUH 103-REC3-118).

### 2.2. Measurement of Thickness of Crestal Cortical Bone at Prospective Dental Implant Sites

**Figure 1:**
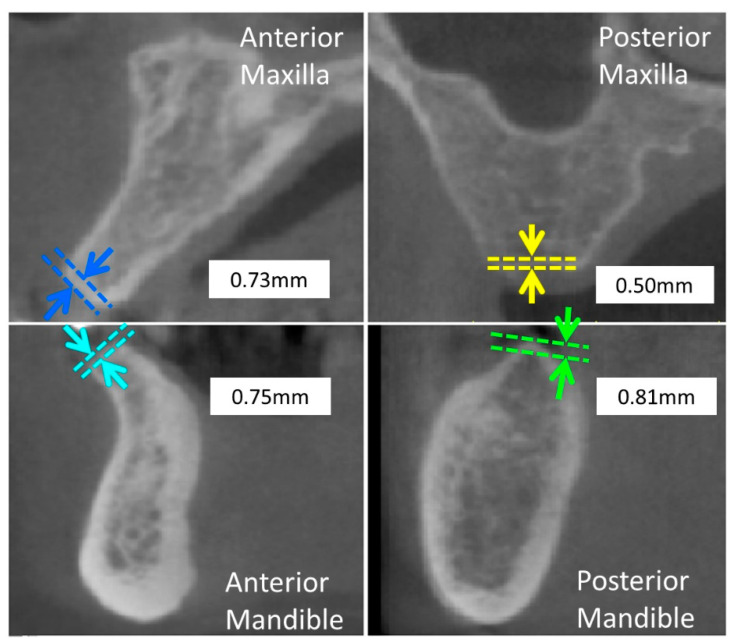
Thickness of crestal cortical bone at the dental implant site for the four jawbone regions, measured using cone-beam computed tomography (CBCT) images.

All CBCT images were imported into Mimics software 15.0 (Materialise, Leuven, Belgium) for measuring the thickness of the crestal cortical bone. Specifically, before the thickness was measured, continual buccolingual images of the mandible and maxilla were created using Mimics’ online reslice function. The central buccolingual image of the prospective dental implant site was then used to measure the thickness of the crestal cortical bone (Figure 1).

### 2.3. Statistical Analysis

All statistical analyses were performed using SPSS 19 (IBM Corporation, Armonk, NY, USA). The mean and standard deviation (SD) of the thickness of the crestal cortical bone were calculated. For both groups, one-way analysis of variance and Tukey’s test for multiple comparisons were used to evaluate differences in the thickness of the crestal cortical bone among the four jawbone regions (anterior maxilla, anterior mandible, posterior maxilla, and posterior mandible). Statistical significance was indicated if *p* < 0.05. Student’s *t-*test was further used to evaluate differences between the younger and older groups.

## 3. Results

**Figure 2:**
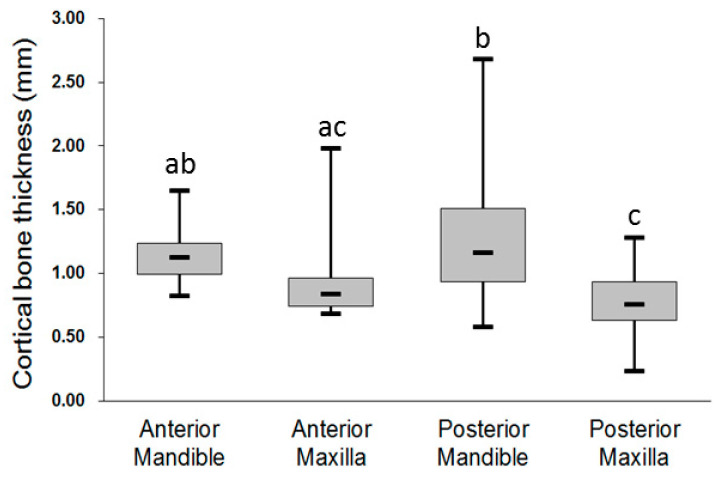
Thickness of the crestal cortical bone at the four jawbone regions for the younger group. Post hoc pairwise comparisons were conducted using Tukey’s test; use of the same letter (a, b, c) indicates no significant difference at the 0.05 level.

For the younger group, the mean thickness of the crestal cortical bone was the highest in the posterior mandible (1.29 ± 0.46 mm), followed by the anterior mandible (1.13 ± 0.20 mm), anterior maxilla (0.89 ± 0.26 mm), and posterior maxilla (0.77 ± 0.24 mm) (Figure 2). However, only the following three comparisons in the mean thickness of crestal cortical bone were statistically significant: anterior mandible (1.13 ± 0.20 mm) > posterior maxilla (0.77 ± 0.24 mm), *p* = 0.003; posterior mandible (1.29 ± 0.46 mm) > anterior maxilla (0.89 ± 0.26 mm), *p* < 0.001; and posterior mandible (1.29 ± 0.46 mm) > posterior maxilla (0.77 ± 0.24 mm), *p* < 0.001.

**Figure 3:**
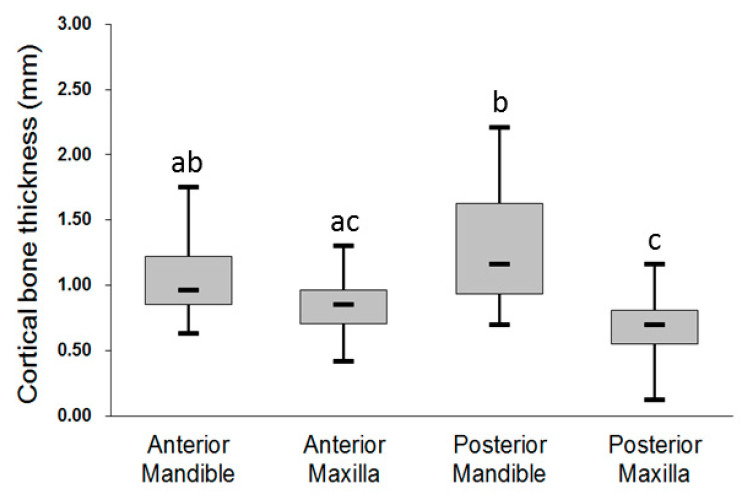
Thickness of the crestal cortical bone at the four jawbone regions for the older group. Post hoc pairwise comparisons were conducted using Tukey’s test; use of the same letter (a, b, c) indicates no significant difference at the 0.05 level.

For the older group, the mean thickness of the crestal cortical bone was the highest in the posterior mandible (1.27 ± 0.40 mm), followed by the anterior mandible (1.08 ± 0.33 mm), anterior maxilla (0.85 ± 0.21 mm), and posterior maxilla (0.66 ± 0.24 mm) (Figure 3). However, only the following three comparisons in the mean thickness of the crestal cortical bone were statistically significant: anterior mandible (1.08 ± 0.33 mm) > posterior maxilla (0.66 ± 0.24 mm), *p* < 0.001; posterior mandible (1.27 ± 0.40 mm) > anterior maxilla (0.85 ± 0.21 mm), *p* < 0.001; and posterior mandible (1.27 ± 0.40 mm) > posterior maxilla (0.66 ± 0.24 mm), *p* < 0.001.

Relative to the younger group, the older group exhibited a thinner mean thickness of crestal cortical bone. However, only in the posterior maxilla region (*p* = 0.008) was the crestal bone significantly thinner (at 14.3%) in the older group than in the younger group (Table 1).

## 4. Discussion

Recently, dental implantation has become commonly used to treat missing teeth. However, the success rate of dental implantation surgery is affected by bone quality and quantity at the dental implant site. A better host bone condition provides greater stability for the dental implant, resulting in enhanced osseointegration and an increased survival rate. Studies have employed computed tomography (CT) or CBCT to investigate cancellous bone density or thickness of crestal cortical bone at prospective dental implant sites. However, it remains uncertain whether being at menopause influences bone density and bone quality at the implant site. This study used CBCT to evaluate the association between being of menopausal age and the thickness of the crestal cortical bone at the dental implant site in the female population. One notable finding was that relative to younger women, older women had lower thickness of the crestal cortical bone in the posterior maxilla region.

The most commonly used method for classifying bone quality and quantity involves a two-dimensional radiograph; this method was proposed by Lekholm Zarb [16] in 1985. This method categorizes jawbones into four categories (Type I to Type IV bones) depending on the thickness of the cortical bone and density of the cancellous bone, with Type I bone having the best bone quality and quantity, and Type IV bones having the poorest bone quality and quantity. Although this classification method is straightforward, it is susceptible to subjective judgements and does not allow quantitative data to be used for classification. Norton and Gamble [17] used CT to measure the Hounsfield units of bones at dental implant sites for bone density evaluation. This method allows quantitative evaluation of bone status at dental implants. In 2009, Vercellotti [18] proposed a new classification method to evaluate cortical and cancellous bones separately. Cortical bone thickness is divided into four categories: 0, 1, 2, and ≥3 mm. Cancellous bone density is divided into the categories of high, medium, and low density. This method enables objective evaluation of bone quality and quantity at dental implant sites. Many scholars have adopted CBCT to measure cancellous bone density [19,20,21] or crestal cortical bone thickness [22,23] at dental implant sites. However, few such studies have investigated the effect of menopause on bone quality and quantity at implant sites.

The increasing popularity of CBCT has prompted its use among dentists in determining jaw bone quality and quantity. Compared with clinical CT, CBCT machines are cheaper, emit lower radiation doses, and have higher voxel resolutions [24,25,26,27]. Therefore, scholars have employed CBCT to measure the jaw’s cortical bone thickness. Sumer et al. [28] indicated the high suitability of CBCTs for measuring palatal cortical bone thickness at the dental implant site; Tsutsumi et al. [29] also noted such a suitability in instances where the cortical bone thickness is three to four times greater than the voxel resolution of CBCT. In this study, among the 340 values for crestal cortical bone thickness, only 16 cases had values less than 450 μm. Therefore, because most measured values of thickness of crestal cortical bone exceeded the 150 μm voxel resolution of CBCT by three times or more, CBCT was a suitable approach for measuring thickness of crestal cortical bone.

Studies have employed CT or CBCT to measure the thickness of the crestal cortical bone at the dental implant site. Miyamoto et al. [14] used CT to undertake such measurements at 225 prospective dental implant sites, reporting a mean thickness of the crestal cortical bone of 1.49 ± 0.34 m and 2.22 ± 0.47 m at the maxilla and mandible, respectively. Using CBCT, Gerlach et al. [13] reported a mean thickness of crestal cortical bone of 2.00 ± 0.15 m at the mandible. Using CT for 75 prospective dental implant sites, Sugiura et al. [15] reported a mean thickness of the crestal cortical bone of 1.5 ± 0.7 m at the posterior mandible. As evident in these measurements, studies have reported different thickness of the crestal cortical bone. Because this study employed CBCT with a voxel resolution of only 150 μm, which is far smaller than the voxel resolutions of previous studies employing CT or CBCT, the thickness of the crestal cortical bone of prospective dental implant sites measured in this experiment was thinner than those in previous studies. Because of the partial volume effect, higher scanning resolutions can result in an overestimation of the thickness value. We conjecture that this is why values measured in previous studies are thicker than those in our study.

The National Health and Nutrition Examination Survey investigated the occurrence rates of low bone mass and osteoporosis among men and women over 50 years old, reporting that osteoporosis was more common in women (i.e., women at postmenopause) than in men [30], demonstrating that women are highly susceptible to osteoporosis at menopause. Osteoporosis increases the incidence of hip bone, spine, and wrist fractures. Many studies have discussed whether osteoporosis results in sustained bone loss in the mandible. Recently, dental implant surgeries have become increasingly popular. The bone quality and quantity at the dental implant site are key factors that determine the success of dental implant surgery, and excellent osseointegration effect of the region surrounding the dental implant is necessary [4]. Several studies have indicated that the success rate of dental implant surgery is affected by osteoporosis status [5,6,7]. Additionally, studies have indicated that women at postmenopause were susceptible to osteoporosis in the jawbone, which potentially influences their chewing function and total number of teeth [3,31]. The current study found that cortical bone thickness differed among the four jawbone regions and between women in the two groups. Although the younger group exhibited higher cortical bone thickness in all regions, only the difference in thickness of the posterior maxilla region (14.3%) was significant. Therefore, dentists must be particularly attentive to bone quality and quantity when inserting dental implants in the posterior maxilla region of older women.

For both groups of our study, thickness of crestal cortical bone were, in descending order, those of the posterior mandible, anterior mandible, anterior maxilla, and posterior maxilla. This result differs from that of studies that have used CT to measure cancellous bone density; their thickness of the crestal cortical bone were, in descending order, those of the anterior mandible, anterior maxilla, posterior mandible, and posterior maxilla [10,11,12]. Among the four jawbone regions, regions with the thickest cortical bone and the highest cancellous bone density are the posterior mandible region and the anterior mandible region, respectively. By comparison, the posterior maxilla region, which is situated at the maxillary sinus position, has the lowest cancellous bone density and thinnest cortical bone among the four regions. In general, the cortical bone influences the initial stability of the dental implant, whereas the cancellous bone determines the potential for osseointegration after the dental implant into the jawbone. Therefore, we suggest including cortical bone thickness and cancellous bone density as independent variables in further research on the bone quality and quantity of prospective dental implant sites.

This study has several limitations. First, because of the retrospective nature of this study, we did not have information regarding patients’ menopause status. Therefore, on the basis of previous studies’ findings [32,33] and the typical menopausal age in Taiwan, we selected 50 years as the menopause age for dividing the patients into groups. Second, the duration of healing between tooth extraction and dental CBCT execution was not obtained due to the fact that this was a retrospective study. Third, prior to the study, no sample size estimation was performed because of the lack of a suitable reference study. However, the current sample size was greater than that of many previous studies that have used CT or dental CBCT to measure jawbone density or cortical bone thickness [9,10,34,35,36,37]. Fourth, because we only employed CBCT scanning to measure the thickness of crestal cortical bone of prospective dental implant sites, we could not measure the stability of dental implants after their implantation into the jawbone. Fifth, this study measured only the crestal cortical bone thickness at the prospective dental implant sites. The cancellous bone density in jawbones was not measured. In addition, similarly to other studies that have used CBCT to measure jawbone quality and quantity, this study did not explore the individual conditions of the patients. Last but not least, all of our participants were of East Asian descent. Thus, whether our findings generalize to other ethnicities requires further study. Furthermore, because this study investigated the association between being of menopausal age and the thickness of the crestal cortical bone at the prospective dental implant site, we did not examine the duration of the women’s menopause, their hormone statuses, or their daily vitamin D and calcium intakes. More comprehensive studies are required to address these limitations.

## 5. Conclusions

For both younger and older women, the regions with the highest and lowest crestal cortical bone thickness are found in the posterior mandible and posterior maxilla regions, respectively. Furthermore, relative to younger women, older women tend to have thinner crestal cortical bone, especially in the posterior maxilla region.

## Figures and Tables

**Table 1:** Sample size, significance, and mean ± SD for the four jawbone regions of the younger and older groups.

Region	Younger Group		Older Group		*p* ^†^
	Number of Patient/Dental Implant Site	Mean ± SD (mm)	Number of Patient/Dental Implant Site	Mean ± SD (mm)	
Anterior maxilla	11/26	0.89 ± 0.26	6/19	0.85 ± 0.21	0.589
Posterior maxilla	18/48	0.77 ± 0.24	30/84	0.66 ± 0.29	0.008 *
Anterior mandible	6/16	1.13 ± 0.20	6/24	1.08 ± 0.33	0.615
Posterior mandible	26/59	1.29 ± 0.46	20/64	1.27 ± 0.40	0.823

^†^ Student’s *t*-test. * Statistical significance (*p* < 0.05).
